# Elucidation of the molecular responses to waterlogging in Jatropha roots by transcriptome profiling

**DOI:** 10.3389/fpls.2014.00658

**Published:** 2014-12-02

**Authors:** Piyada Juntawong, Anchalee Sirikhachornkit, Rachaneeporn Pimjan, Chutima Sonthirod, Duangjai Sangsrakru, Thippawan Yoocha, Sithichoke Tangphatsornruang, Peerasak Srinives

**Affiliations:** ^1^Special Research Unit in Microalgal Molecular Genetics and Functional Genomics, Department of Genetics, Faculty of Science, Kasetsart UniversityBangkok, Thailand; ^2^National Center for Genetic Engineering and BiotechnologyPathumthani, Thailand; ^3^Department of Agronomy, Faculty of Agriculture at Kamphaeng Saen, Kasetsart UniversityNakhon Pathom, Thailand

**Keywords:** *Jatropha curcas*, genomics, low oxygen, ERFs, transcriptional control, RNA-seq

## Abstract

Jatropha (*Jatropha curcas*) is a promising oil-seed crop for biodiesel production. However, the species is highly sensitive to waterlogging, which can result in stunted growth and yield loss. To date, the molecular mechanisms underlying the responses to waterlogging in Jatropha remain elusive. Here, the transcriptome adjustment of Jatropha roots to waterlogging was examined by high-throughput RNA-sequencing (RNA-seq). The results indicated that 24 h of waterlogging caused significant changes in mRNA abundance of 1968 genes. Comprehensive gene ontology and functional enrichment analysis of root transcriptome revealed that waterlogging promoted responses to hypoxia and anaerobic respiration. On the other hand, the stress inhibited carbohydrate synthesis, cell wall biogenesis, and growth. The results also highlighted the roles of ethylene, nitrate, and nitric oxide in waterlogging acclimation. In addition, transcriptome profiling identified 85 waterlogging-induced transcription factors including members of AP2/ERF, MYB, and WRKY families implying that reprogramming of gene expression is a vital mechanism for waterlogging acclimation. Comparative analysis of differentially regulated transcripts in response to waterlogging among Arabidopsis, gray poplar, Jatropha, and rice further revealed not only conserved but species-specific regulation. Our findings unraveled the molecular responses to waterlogging in Jatropha and provided new perspectives for developing a waterlogging tolerant cultivar in the future.

## Introduction

Waterlogging is an adverse abiotic stress that can heavily damage crop production worldwide. The condition can be defined as the saturation of soils with water. Waterlogging occurs during the heavy rainy season in lowland areas, including Thailand. Due to the limited diffusion of gas under water, waterlogging creates low oxygen (hypoxia) environments in the root areas, causing a shortage of ATP from the inhibition of oxidative phosphorylation. Upon experiencing long-term waterlogging stress (WS), stomatal closure can lead to impaired root hydraulic conductivity, thereby reducing the photosynthetic rate and the nutrient and water uptake of the plant.

*Jatropha curcas* (common names: Jatropha, physic nut, and purging nut) is a promising crop for the generation of biodiesel. Jatropha seeds contain between 40 and 50% of high quality oil (Basha and Sujatha, 2009). Based on dominant marker analysis among worldwide populations, Jatropha possesses a narrow genetic base (Popluechai et al., 2009). Indigenous to South America, Jatropha is drought tolerant, however, the species is highly sensitive to waterlogging. Gimeno et al. (2012) reported that 10 days of waterlogging caused an approximately 30% reduction in Jatropha biomass. Moreover, a decrease in leaf photosynthetic rate and a decline in carbohydrate concentration in both leaves and roots were observed in waterlogged Jatropha (Gimeno et al., 2012). Despite these lines of evidence, the molecular mechanism underlying the waterlogging response in Jatropha remains unknown.

In recent years, studies in Arabidopsis (*Arabidopsis thaliana*), gray poplar (*Populus × canescens*), cotton (*Gossypium hirsutum*), and rice (*Oryza sativa*) have revealed that low oxygen can cause drastic changes in transcription, translation, and metabolite levels (Branco-Price et al., 2008; Kreuzwieser et al., 2009; Mustroph et al., 2009; Narsai et al., 2009; Christianson et al., 2010; Lee et al., 2011; Juntawong et al., 2014). The low oxygen condition results in limited ATP production and negatively affects cellular energy status. Therefore, a key feature for the acclimation to low oxygen environment is to activate genes encoding proteins and enzymes for anaerobic fermentation, glycolysis, transcription factors, and signaling pathways in order to allow biological and physiological adjustments to the low oxygen conditions (Bailey-Serres et al., 2012). While several published studies of model plants have provided some fundamental clues, quite a few studies were performed in hypoxic chambers, in which the entire plants were subjected to low oxygen conditions, grown on artificial media, or subjected to complete darkness. These conditions would not imitate the impact observed in plants exposed to soil waterlogging. Recently, high-throughput sequencing technology has been used as a powerful tool for genomic analysis in both model and non-model species. This method can provide quantitative descriptions of gene expression at the genome-scale level, accompanying high accuracy, low background noise, and large dynamic ranges. High-throughput transcriptome analysis could help provide a basic understanding of the molecular responses to waterlogging in Jatropha.

The goal of this study is to provide new insights into the molecular responses of Jatropha to waterlogging. Here, we profiled transcriptome changes in Jatropha roots subjected to 24 h of waterlogging, using high-throughput sequencing by the Ion Proton platform. Bioinformatic analysis of transcriptome data was performed to allow identification and functional annotation of differentially expressed genes (DEGs). Additionally, we comparatively analyzed waterlogging transcriptomes from Arabidopsis, Jatropha, gray poplar and rice to identify conserved and species-specific responses. Lastly, we discussed the use of genetic engineering to target some candidate genes for generation of waterlogging-tolerant Jatropha.

## Materials and methods

### Plant materials and stress conditions

*Jatropha curcas* seeds (cv. “Chai Nat”—a Thai local variety) were germinated in soil containing 50% (v/v) peat moss, 25% (v/v) perlite, and 25% (v/v) coconut fiber with a daily water supply. Plants were grown outdoors between July and August of 2013 and 2014 at Kasetsart University, Bang Khen campus. Thirty day-old, six-leaf-stage plants were used in the waterlogging treatment. In brief, plant pots were placed in plastic containers filled with tap water. The level of water was set at 3 cm above the soil. WS began at mid-day and continued for 24 h. For the control, non-treated plants were placed in a container with no water. Root tissue was harvested at the end of the treatment, immediately placed in liquid nitrogen, ground into a fine powder, and kept at −80°C.

### RNA extraction, library preparation, and sequencing

Total RNA was extracted with TRIzol reagent (Invitrogen), according to the manufacturer's protocol. Total RNA samples were subjected to DNase treatment and RNA cleanup by a GF-1 RNA extraction kit (Vivantis). Poly(A)^+^ mRNAs were isolated from the total RNAs by using an Absolutely mRNA purification kit (Agilent). Jatropha mRNA (200 ng) was used to construct a sequencing library, by using a Ion Total RNA seq kit (Life Technologies). For each treatment, two independent biological replicates were sequenced on a Ion Proton sequencer (Life Technologies). FASTQ files were obtained with the base caller provided by the instrument and subjected to quality control filtering and 3′end trimming using the FASTX-toolkit (http://hannonlab.cshl.edu/fastx_toolkit/index.html) included in the Torrent Suite™ Software (Life Technologies). The preprocessed reads with a minimum of 35 nt and a quality value of 17, were deposited in the NCBI GEO database under the accession number GSE57428.

### Transcriptome analysis

FASTQ reads were mapped to the *J. curcas* genome release 4.5, downloaded from http://www.kazusa.or.jp/jatropha/ (Sato et al., 2011; Hirakawa et al., 2012) with TMAP mapping program (https://github.com/iontorrent/TMAP), using the default settings and “mapall” command. A binary format of sequence alignment files (BAM) was generated and subsequently used to create read count tables using HTseq library (Anders et al., 2014) for differential gene expression analysis. Statistical analysis of DEGs was calculated in the R environment (R Development Core Team, 2005), by using a generalized linear model (GLM) approach from the edgeR package (Robinson et al., 2010). Genes with the count per million (CPM) values ≥ 1 in at least two library samples were included in this analysis (Supplementary Table S1). In brief, the calcNormFactors function was applied for data normalization using the trimmed mean of M-values (TMM) method. Subsequently, the GLMTagwiseDisp function was used to generate the estimated tagwise dispersion. Next, the glmFit function was applied to fit the negative binomial GLM for each tag. Finally, the glmLTR function was applied to carry out the likelihood ratio test. Significant DEGs were filtered, based on false discovery values (FDR < 0.05).

Assignment of the DEGs into functional bins and the creation of a mapping file from the Jatropha genome were performed using the Mercator pipeline for automated sequence annotation (Lohse et al., 2013). The inputs for the Mercator were Jatropha protein sequences supplied by http://www.kazusa.or.jp/jatropha/. This analysis was performed with a blast score cutoff > 80. For visualization, the MapMan (Thimm et al., 2004) and the PageMan (Usadel et al., 2006) programs were used.

Gene ontology (GO) enrichment analysis was performed in the R environment. Firstly, the GO terms for each gene were derived from homology searching of plant protein databases using GOANNA with an *e*-value cutoff < E^−10^ (McCarthy et al., 2006). Subsequently, a gene annotation file was generated. GO term enrichment analysis in up or down regulated DEGs was performed by the GOHyperGALL function (Horan et al., 2008). Significant GO terms were filtered based on the adjusted *p*-value < 0.05.

### Identification of Jatropha AP2/ERF genes

For mining of AP2/ERF genes from the Jatropha genome, the hidden Markov model (HMM) profile of the AP2/ERF superfamily was extracted from the Pfam and used to search Jatropha protein sequences.

To perform phylogenetic analysis comparing Jatropha and Arabidopsis AP2/ERF genes, the amino acid sequences of the Arabidopsis AP2/ERF genes were downloaded from the TAIR database (http://www.arabidopsis.org/). A multiple alignment was performed using the MUSCLE alignment in the MEGA5 software (Tamura et al., 2011). A phylogenetic tree of AP2/ERF genes was constructed by the Neighbor Joining method, with a bootstrap number set to 1000 replicates. A list of predicted Jatropha AP2/ERF genes can be found in Supplementary Table S2.

### Quantitative real-time PCR

cDNA was synthesized from 1.2 μg of total RNAs using oligo(dT)20 and superscript III reverse transcriptase (Invitrogen), according to the manufacturer's protocol. Real-time PCR was assayed in a 20 μL reaction, containing KAPA SYBR FAST qPCR master mix (KAPABIOSYSTEMS) using the Stratagene Mx3000P real-time PCR system (Agilent Technologies). Amplification specificity was validated by melt-curve analysis at the end of each PCR experiment. Relative gene expression was calculated using the comparative ΔΔcT method (Livak and Schmittgen, 2001). *Ubiquitin protein* (*UBC; Jcr4S00238.120*) was used as a normalization control. Primer sequences and annealing temperatures were listed in Supplementary Table S3.

### Analysis of publicly available microarray data

Publicly available microarrays were downloaded from the Gene Expression Omnibus (GEO) database (details listed in Supplementary Table S4). Microarray data were normalized using the Robust Multi-chip Average (RMA) method. Differential expression analysis was performed on RMA-normalized data. False discovery rates (FDR) for significant differences between genes in each comparison were generated using *p*-value distributions (Smyth, 2004). For each comparison, DEGs were selected based on criteria of FDR < 0.05 and the absolute values of log_2_ fold change (logFC) ≥ 1.

### Ortholog identification

Orthologs among Jatropha, Arabidopsis, rice, and poplar were identified based on protein sequence homology using ORTHOMCL (OMCL) version 1.4 (Li et al., 2003). The inflate number used to perform this analysis was set to 1.2. Protein sequences used in this step were downloaded from Jatropha, Arabidopsis, rice, and poplar genome databases (ftp://ftp.kazusa.or.jp/pub/jatropha/JAT_r4.5.aa.gz, ftp://ftp.arabidopsis.org/home/tair/Sequences/blast_datasets/TAIR10_blastsets/TAIR10_pep_20110103_representative_gene_model_updated, ftp://ftp.jgi-psf.org/pub/compgen/phytozome/v9.0/Osativa/annotation/Osativa_204_protein_primaryTranscriptOnly.fa.gz, and ftp://ftp.jgi-psf.org/pub/compgen/phytozome/v9.0/Ptrichocarpa/annotation/Ptrichocarpa_210_protein_primaryTranscriptOnly.fa.gz, respectively). A list of OMCL clusters can be found in Supplementary Table S5.

### Analysis of leaf chlorophyll content

Chlorophyll content was measured using the atLEAF+ chlorophyll meter (FT Green LLC, Wilmington, DE). The youngest fully expanded leaves of approximately 30 day-old plants were measured three times and the averages were used in subsequence analysis. Six plants were analyzed for each time point. The total chlorophyll content of the leaves was obtained by converting the atLEAF+ values in SPAD using an online webtool: http://www.atleaf.com/SPAD.aspx.

### Carbohydrate assay

One hundred mg of frozen root tissue was used to quantify the total carbohydrate content using a method described by Sadasivam and Manickam (2007). Non-structural carbohydrates were extracted and hydrolyzed by adding 5 mL of 2.5 N HCl and incubated in a boiling water bath for 3 h. The extract was neutralized by adding 0.75 g of Na_2_CO_3_. The anthrone method was used to determine total carbohydrate content relative to a standard series of glucose. In brief, the extract (300 μL) and distilled water (700 μL) were mixed with 4 mL of 0.14% (w/v) anthrone solution in 95% H_2_SO_4_, incubated in a boiling water bath for 8 min, and rapidly cooled on ice. The absorbance was quantified at 630 nm.

## Results

### Waterlogging stress triggered transcriptome readjustment in Jatropha roots

In our observation, we found that long-term waterlogging in young Jatropha seedlings resulted in leaf chlorosis (Supplementary Figure S1A) and reduction of total root carbohydrate content (Supplementary Figure S1B). To determine the molecular responses of Jatropha to waterlogging, six-leaf-stage plants (30-days old) were tested by applying 24 h of waterlogging. First, we examined the response of Jatropha leaves and roots to waterlogging by using the semi-quantitative reverse-transcription PCR. We found waterlogging induced the expression of low oxygen responsive marker genes, *alcohol dehydrogenases* (*ADHs*) and *pyruvate decarboxylase* (*PDC*) in roots, but not in leaves (Supplementary Figure S2). Therefore, our transcriptome analysis was focused only on the response in root organs.

To compare the effects of waterlogging on the Jatropha roots, we quantitatively profiled transcriptome from WS and non-stress (NS) samples. Over 4 million reads were obtained for each of the two independent biological replicates. The majority of reads (93–96%) mapped to the Jatropha genome (Supplementary Figure S3A). Biological replicate transcriptome data were highly correlated as shown by Pearson's correlation coefficient of CPM values (*r* = 0.93 and 0.85 from NS and WS libraries, respectively; Supplementary Figure S3B). A multi-dimension scaling plot of the gene expression data demonstrated that samples were clearly separated by treatments (Supplementary Figure S3C).

**Figure 1:**
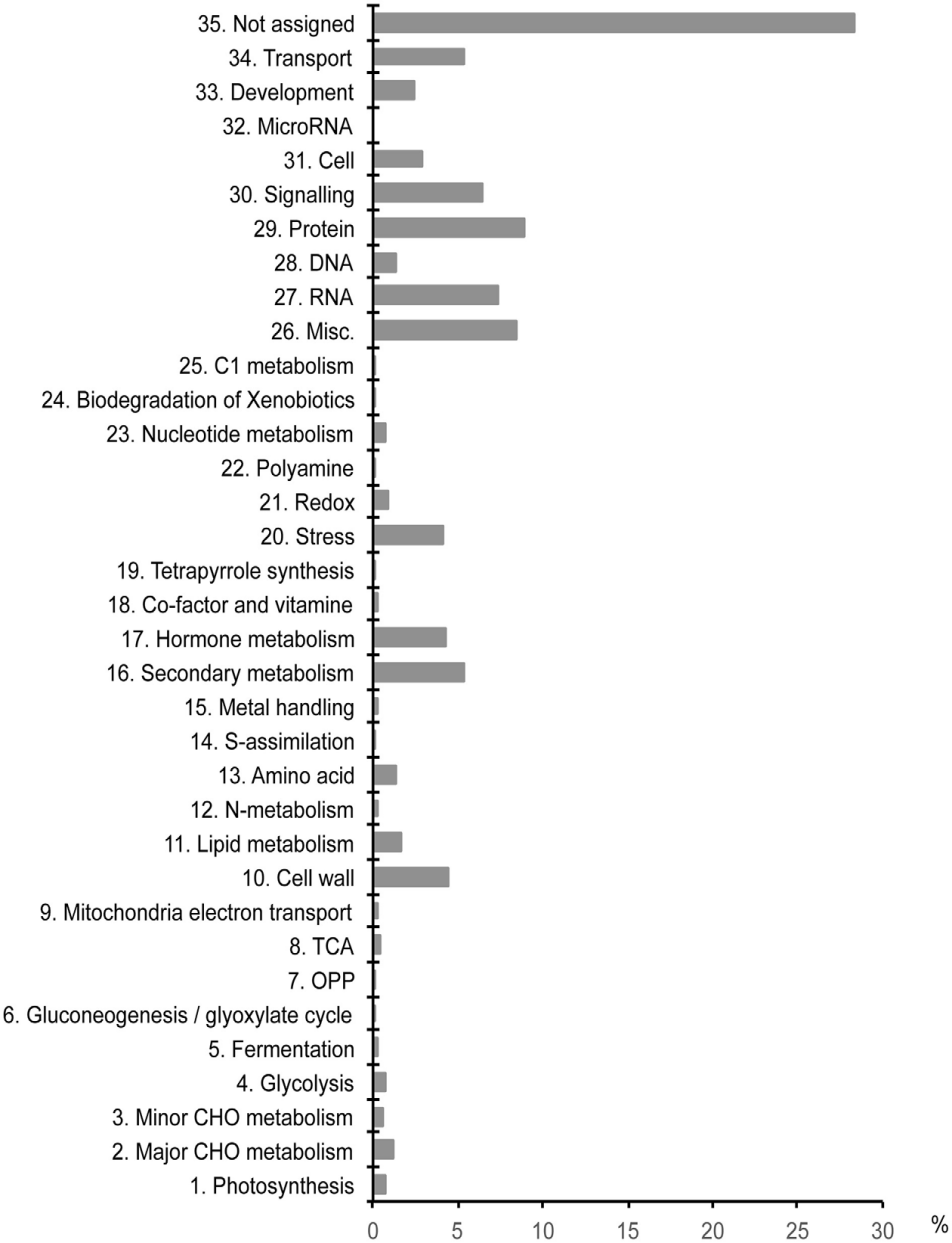
**Functional classification of waterlogging-responsive DEGs**. DEGs (1968 genes) were assigned into 35 functional bins using the Mercator annotation pipeline.

Transcriptome analysis identified 1968 DEGs with significant changes in expression evaluated by the false discovery rate (FDR < 0.05) (Supplementary Table S1). Of these, 931 genes (47%) were up-regulated and 1037 genes (53%) were down-regulated. Assignment of the DEGs to functional bins, using a well-established Mercator pipeline (Lohse et al., 2013), categorized the DEGs into 35 functional bins (Figure 1; Supplementary Table S1). Approximately 28% of the DEGs fell into a “not-assigned” bin. Most of the genes in this bin were annotated as “short,” “partial,” and “transposable element (TE)” by Sato et al. (2011) and Hirakawa et al. (2012). Nevertheless, the genes that related to cell wall, secondary metabolism, hormone metabolism, stress, RNA, protein, signaling, and transport were abundant in the DEG dataset.

**Figure 2:**
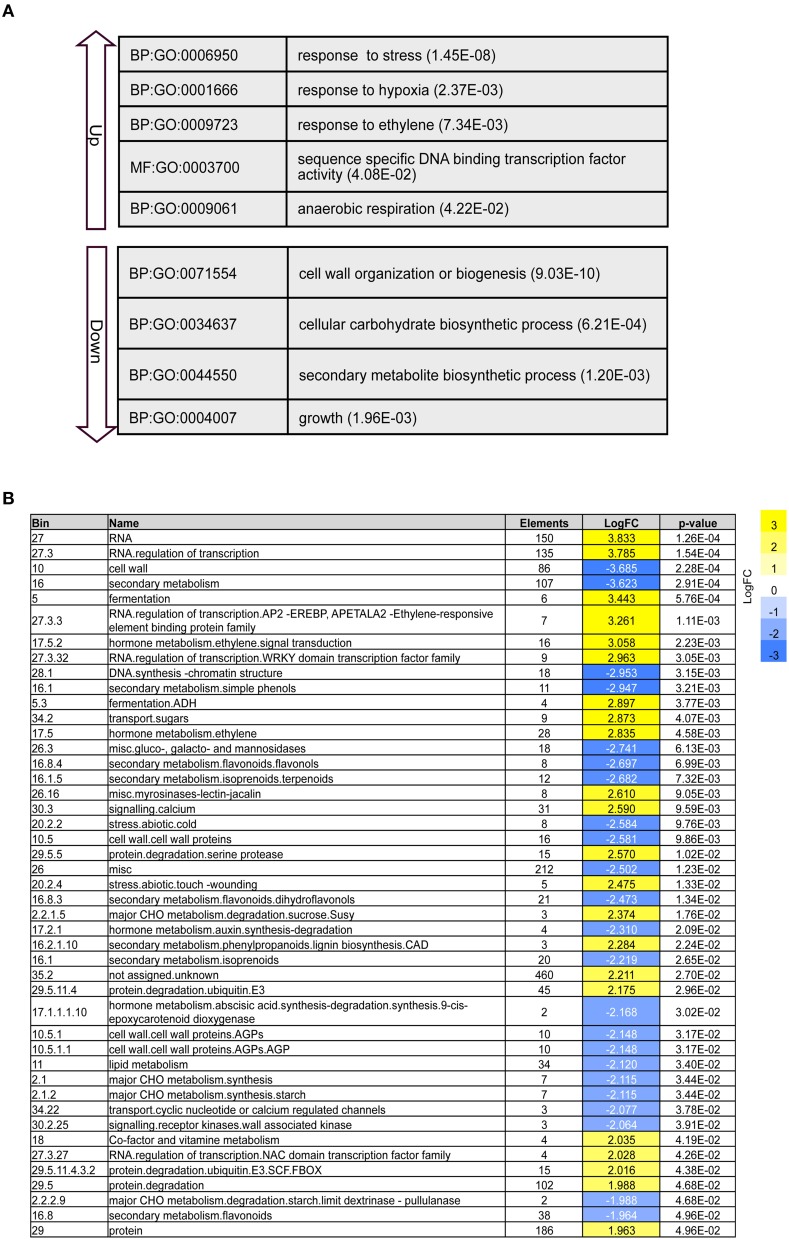
**Waterlogging reprograming transcriptome of Jatropha roots. (A)** Selected gene ontology (GO) categories (*p*-values calculated by GOHyperG) from up-regulated (Up) and down-regulated (Down) groups are shown. **(B)** PAGEMAN enrichment analysis with significant up/down-regulation (Wilcoxon rank sum test; *p*-value < 0.05).

To further evaluate the response to waterlogging, we considered the biological function of the DEGs that were co-regulated in response to waterlogging. GO enrichment analysis was performed with the up-regulated or down-regulated DEGs. Co-regulation of DEGs with similar functions was observed (Figure 2A; Supplementary Table S1). The up-regulated DEGs were involved in the response to stress (adjusted *p*-value: 1.45E-08), response to hypoxia (adjusted *p*-value: 2.37E-03), and response to ethylene (adjusted *p*-value: 7.34E-03). In addition, we also observed enrichment of genes encoding transcription factors (adjusted *p*-value: 4.08E-02) following WS. The down-regulated DEGs were enriched for cell wall organization or biogenesis (adjusted *p*-value: 9.03E-10), cellular carbohydrate biosynthetic process (adjusted *p*-value: 6.21E-04), secondary metabolite biosynthetic process (adjusted *p*-value: 1.20E-03), and growth (adjusted *p*-value: P1.96E-03).

To confirm the results from the GO enrichment analysis, we performed functional enrichment analysis using PAGEMAN tools (Usadel et al., 2006) (Figure 2B). The PAGEMAN results were highly correlated with those derived from the GO enrichment analysis. In fact, results from PAGEMAN further demonstrated that DNA synthesis/chromatin structure related genes were down-regulated (*p*-value: 3.15E-03). On the other hand, the expression of the genes involved in protein degradation, calcium signaling, and sugar transport were enhanced (*p*-value: 4.68E-02, 9.59E-03, and 4.07E-03, respectively). Altogether, the GO enrichment and PAGEMAN results confirmed that waterlogging caused global gene expression reprogramming in Jatropha roots.

### Differential regulation of genes associated with anaerobic fermentation and glycolysis

**Figure 3:**
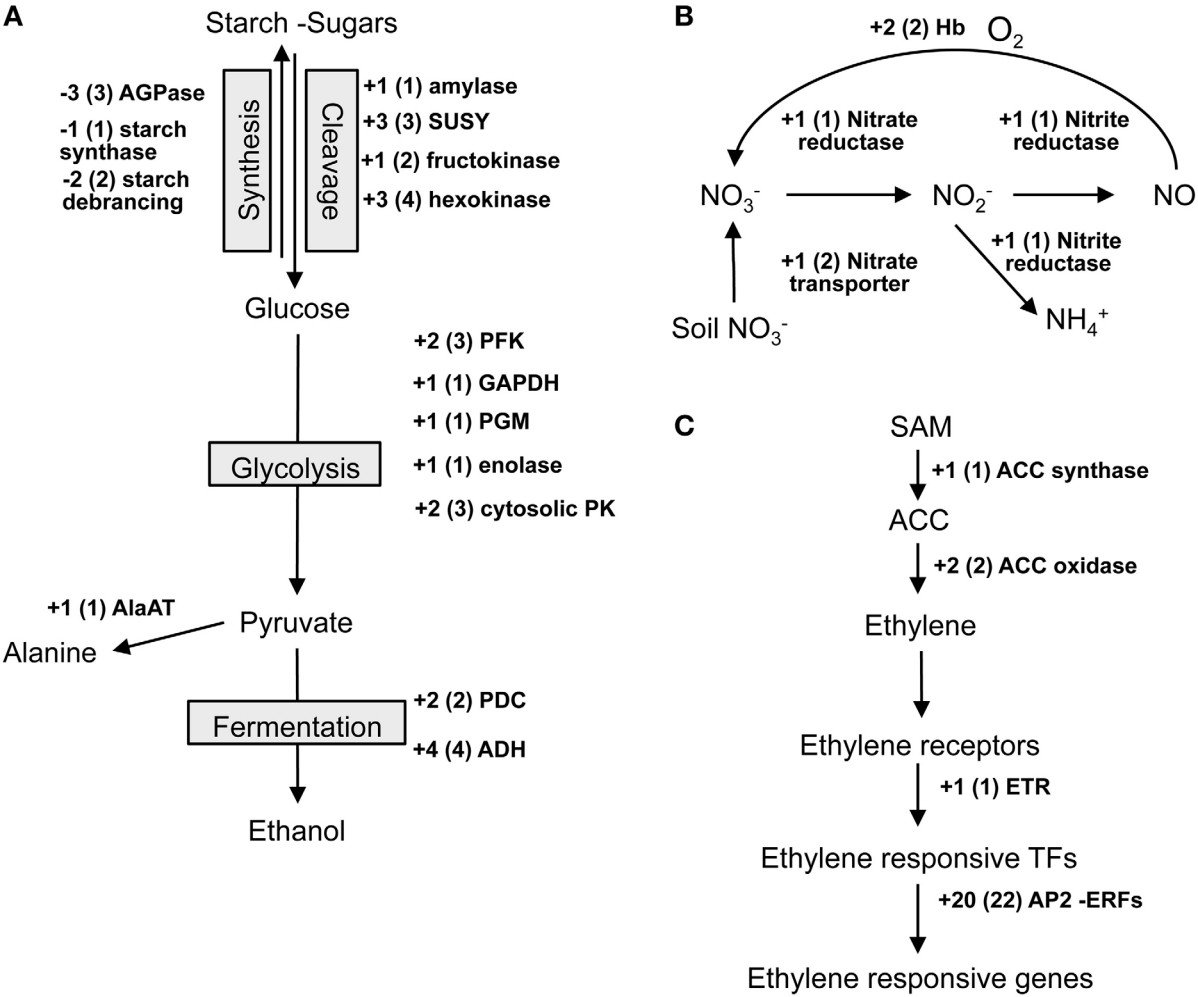
**Waterlogging caused differential expression of transcripts encoding proteins involved in energy, nitrate, and ethylene metabolisms. (A)** Starch metabolism (Starch), glycolysis, and fermentation **(B)** Nitrate metabolism **(C)** Ethylene production and perception. A plus sign represents induction and a minus sign represents reduction. Numbers in brackets represent numbers of DEGs found in this analysis. Data can be found in Supplementary Table S1.

Because the low oxygen conditions could promote glycolysis and fermentation, we further examined regulation of the DEGs encoding central carbon metabolism enzymes (Figure 3A; Supplementary Table S1). We found that waterlogging promoted the expression of the genes involved in sugar and starch cleavage [e.g., *alpha-amylase* and *sucrose synthase* (*SUSY*)]. In contrast, waterlogging inhibited the accumulation of mRNAs encoding for enzymes controlling starch synthesis [e.g., *ADP-glucose pyrophosphorylase* (*AGPase*) and *starch synthase*]. Moreover, the expression of the genes associated with glycolysis was up-regulated by waterlogging [e.g., *phosphofructokinase* (*PFK*), *pyruvate kinase* (*PK*), and *glyceraldehyde phosphate dehydrogenase* (*GAPDH*)]. As expected, an increase in the expression of fermentative genes (*PDC* and *ADH*) was evident. Furthermore, the expression of *alanine transaminase* (*AlaAT*), an enzyme responsible for conversion of pyruvate to alanine, was found up-regulated in our analysis. Overall the results revealed that waterlogging promoted catabolism of carbohydrates and a switch from oxidative to anaerobic respiration in Jatropha roots.

### Differential regulation of genes involved in nitrate metabolism and nitric oxide production

Changes in nitrate metabolism were observed upon waterlogging in several plant species (Horchani et al., 2010; Oliveira et al., 2013; Oliveira and Sodek, 2013). Therefore, we evaluated the regulation of the genes involved in nitrate metabolism and found that a set of genes was up-regulated in response to waterlogging in Jatropha roots (Figure 3B; Supplementary Table S1). These included genes encoding nitrate reductase (NR), a key enzyme responsible for conversion of nitrate (NO^−^_3_) to nitrite (NO^−^_2_) and nitric oxide (NO), nitrite reductase (NIR), an enzyme responsible for reduction of NO^−^_2_ to ammonium and conversion of NO^−^_2_ to NO, and NO^−^_3_ transporter. Besides this, waterlogging induced the expression of *non-symbiotic hemoglobins* (*nsHbs*; Supplementary Table S1), which have been found to be associated with NO removal (Heckmann et al., 2006). These results suggest the regulation of NO^−^_3_ metabolism and that the modulation of endogenous NO levels might be important for waterlogging acclimation in Jatropha.

### Differential regulation of genes related to ethylene synthesis and perception

**Figure 4:**
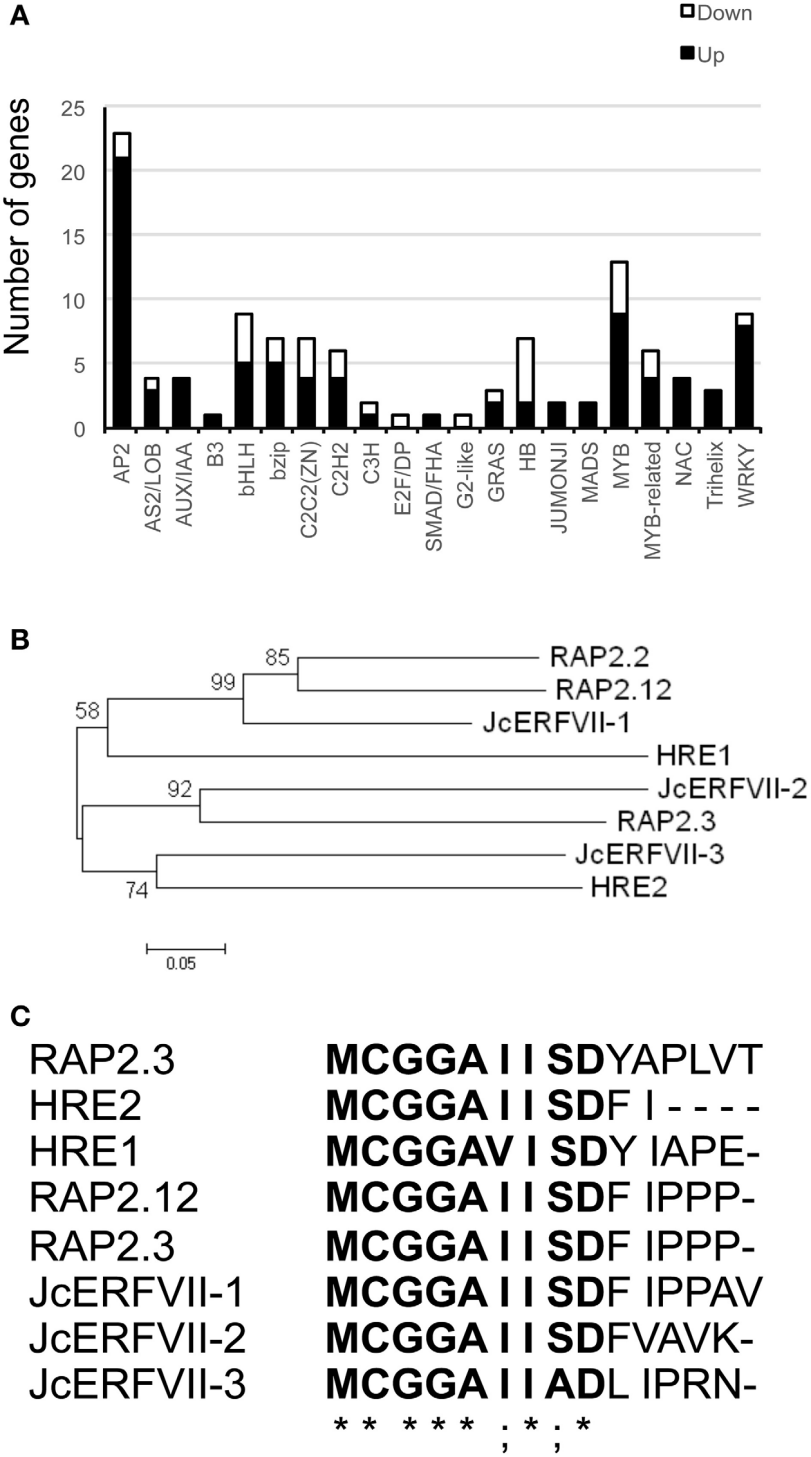
**The group-VII ERFs of Jatropha. (A)** Graphical representation of waterlogging-regulated transcription factors based on their assigned protein families. “Up” and “Down” represent up-regulation and down-regulation in this analysis. **(B)** Phylogenetic tree of group-VII ERF proteins. The full-length protein sequences were analyzed with a Neighbor-joining method. Numbers above branches represent the bootstrapped value from 1000 replicates. **(C)** Multiple sequence analysis of N-terminal sequences of group-VII ERFs. Bold letters indicate a conserved motif at the N-terminus initiated with Met_1_-Cys_2_, as identified by multiple sequence alignment. Asterisks and semi-colons indicate identical and conserved substitution, respectively.

Accumulation of a plant stress hormone, ethylene, in response to low oxygen has been reported (Voesenek et al., 1993; Vriezen et al., 1999; Rzewuski et al., 2007; Hinz et al., 2010). Our analysis demonstrated the differential regulation of DEGs encoding for proteins functioning in ethylene synthesis and response (Figure 3C; Supplementary Table S1). The expression of two key-genes controlling ethylene synthesis, *1-aminocyclopropane-1-carboxylate* (*ACC*) *synthase* (*ACS*) and *ACC oxidase* (*ACO*), was up-regulated by waterlogging. In addition, we found that waterlogging induced accumulation of transcripts encoding for ethylene receptor (ETR). Interestingly, several genes encoding for ERF (ethylene responsive factors) transcription factors (TFs) were up-regulated by waterlogging (Figure 4A). These marks highlighted the roles of ethylene in acclimation to waterlogging in Jatropha.

### Differential regulation of genes encoding TFs

Since low oxygen induced TFs could mediate the expression of anaerobic responsive genes, we analyzed for TFs that were differentially regulated by waterlogging. One-hundred and fifteen waterlogging-regulated TFs were classified based on their assigned protein families (Figure 4A; Supplementary Table S1). Of these, 85 TFs were up-regulated and 30 TFs were down-regulated. Remarkably, TFs accounted for nearly six percent of the DEGs analyzed in this study. ERFs (21 genes), MYBs (9 genes), and WRKYs (8 genes) were mostly pronounced from the up-regulated group, whereas Homeobox TFs (HBs; 5 genes) were mostly noticeable from the down-regulated group. Recent studies on Arabidopsis demonstrated that group-VII ERFs are modulators of the anaerobic response under low oxygen conditions (Gibbs et al., 2011; Licausi et al., 2011). Therefore, we examined the expression of the group-VII ERFs in Jatropha. Firstly, we searched the Jatropha genome using the HMM profile of the Apetalla2 (AP2)/ERF domain in order to identify all AP2/ERF genes. As a result, 133 putative AP2/ERF genes were identified. Secondly, we performed sequence alignment and phylogenetic analyses to compare Arabidopsis and Jatropha ERFs. Finally, 14 groups of AP2/ERF genes, including 11 ERF subgroups, AP2, RAV, and soloist were successfully identified (Supplementary Table S2). This analysis revealed that the Jatropha group-VII ERFs comprised three members, all possessing a conserved N-terminal domain [NH_2_-MCGGAII(A/S)D] (Figures 4B,C). Remarkably, two of them (*Jcr4S00982.160* and *Jcr4S01651.60*; *JcERFVII-2* and *JcERFVII-3*, respectively) were up-regulated in our transcriptome analysis (Supplementary Table S1). Together, these data implicated the essence of transcriptional regulation in response to waterlogging.

### Validation of differentially regulated genes by quantitative real-time PCR

**Figure 5:**
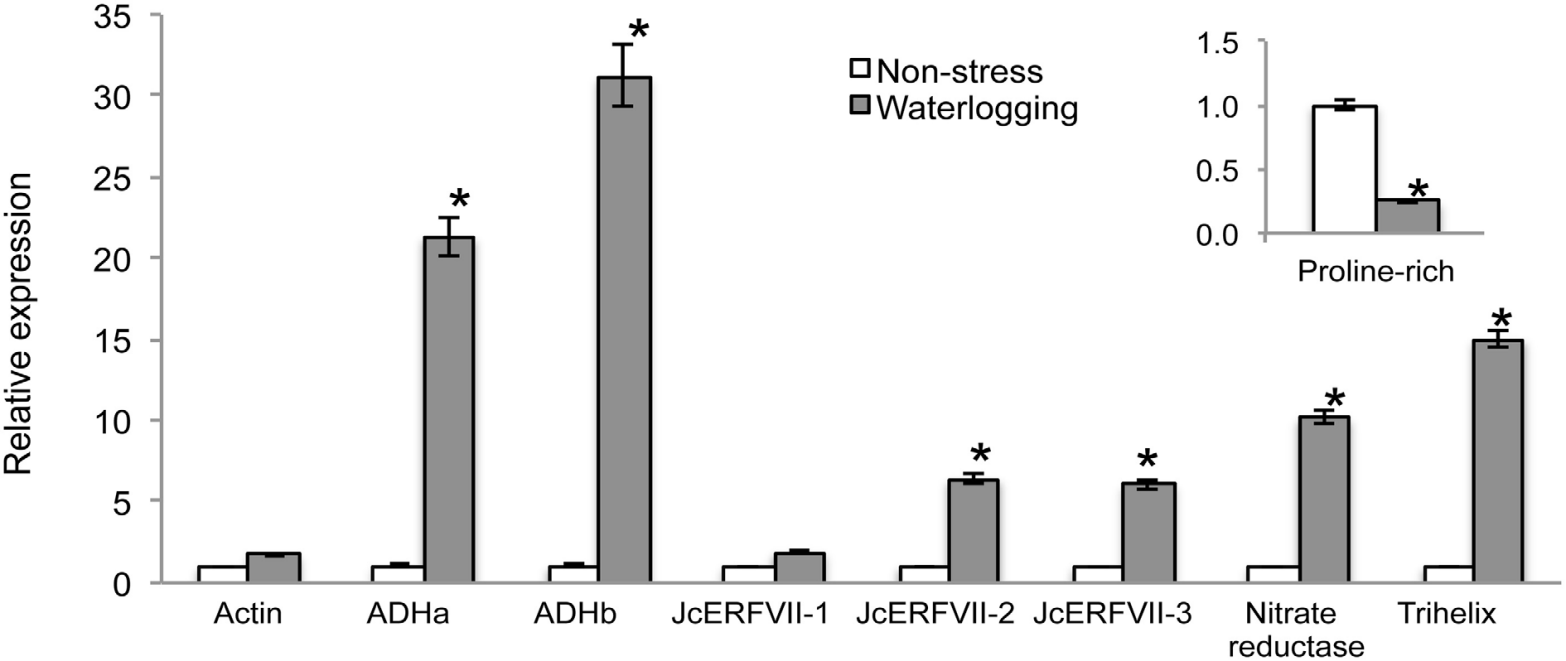
**Quantitative real-time PCR validation of transcriptome data for selected genes**. Relative expression was normalized to the abundance of *Ubiquitin* (*UBC*; *Jcr4S00238.120*). Data represent mean ± SE (*n* = 3). ^*^p-value < 0.01 (*t*-test).

To verify the transcriptome data, 10 genes were selected for analysis by quantitative real-time PCR to determine their relative expression in response to WS. These selected genes included 6 waterlogging-induced DEGs [*ADHa, ADHb, JcERFVII-2, JcERFVII-3, NR*, and *trihelix TF* (*trihelix*)], two non-DEGs with limited changes in response to waterlogging (*actin* and *JcERFVII-1*), one waterlogging-repressed DEG (*proline-rich*), and one control for fold change analysis (*UBC*). Real-time PCR results of the same samples used for RNA-seq clearly confirmed that waterlogging induced the accumulation of *ADHa, ADHb, JcERFVII-2, JcERFVII-3, NR*, and *trihelix* but not *actin* and *JcERFVII-1* (Figure 5). By contrast, mRNA accumulation of *proline-rich*, a gene encoding a cell-wall protein, was clearly reduced upon waterlogging (Figure 5). These data provided additional support that transcriptome analysis by RNA-seq could be used to quantify genome-scale gene expression in the roots of waterlogged Jatropha.

### Comparative analysis of transcriptome response to waterlogging in arabidopsis, Jatropha, gray poplar and rice

**Figure 6:**
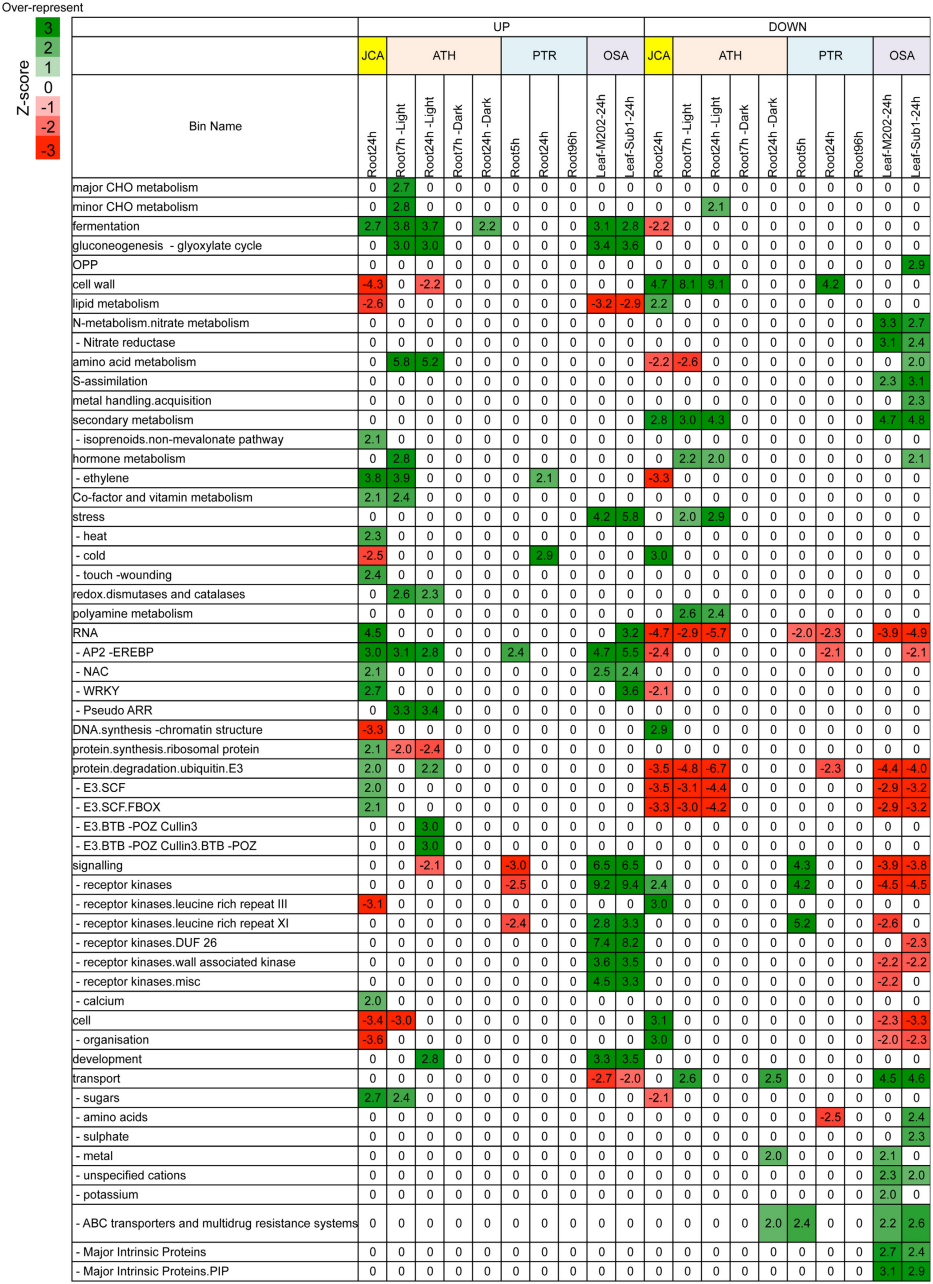
**Overview of transcriptome response for selected functional categories to waterlogging or submergence in Arabidopsis (ATH), Jatropha (JCA), gray poplar (PTR), and rice (OSA)**. PAGEMAN analysis of the gene expression data (|log_2_ fold change| > 1; FDR < 0.05). Statistical analysis of over-represented functional bins was carried out using Fisher method. Z-scores indicate over/under representation (Numbers indicate z-scores; Green, over-represented; Red, under-represented). Data used to generate this figure can be found in Supplementary Table S5.

The variation in tolerance to the low oxygen conditions among plant species has been reported (Bailey-Serres and Voesenek, 2008). In order to identify common or specific changes in transcriptional regulation between waterlogging intolerant Arabidopsis and Jatropha and tolerant gray poplar and rice, we first analyzed public microarray data to identify DEGs in response to waterlogging or submergence in Arabidopsis, gray poplar and rice (Supplementary Table S4). Additionally, we performed a PAGEMAN over-representation analysis (ORA) using Fisher's exact test and a cut-off value of two to obtain an overview of comparative comparison. PAGEMAN ORA analysis allowed comparison of genes with similar functions. We observed an over-represented induction of genes encoding for anaerobic fermentation in Arabidopsis, Jatropha, and rice (Figure 6; Supplementary Table S5). In addition, AP2/ERF DNA binding TFs were up-regulated in all species. The down-regulation of genes involved in cell wall processes and secondary metabolism was common in both tolerant and intolerant species (Figure 6; Supplementary Table S5).

Additionally, we attempted to compare changes in expression of orthologs in the genomes of Arabidopsis, Jatropha, gray poplar and rice. OrthoMCL (OMCL) cluster analysis using a Markov Cluster algorithm was applied in order to group orthologs (Li et al., 2003). This analysis identified 19,103 OMCL clusters (Supplementary Table S5). Over 7000 OMCL clusters were differentially regulated upon waterlogging (Supplementary Figure S4). We found that using a list of 49 core hypoxia genes reported to be up-regulated in response to low oxygen stress in shoots and roots of Arabidopsis seedlings (Mustroph et al., 2009), we were able to identify 37 OMCL clusters among Arabidopsis, poplar, Jatropha, and rice (Supplementary Figure S5). Of these, 30 OMCL clusters were induced in at least two species. OMCL236 (wound-responsive proteins), OMCL442 (pyruvate decarboxylase), OMCL450 (alcohol dehydrogenase), and OMCL1101 (ethylene receptor) were induced in all species. We further compared the expression of OMCL clusters involved in carbohydrate cleavage, glycolysis, and fermentation and, in general, found up-regulation of OMCL clusters in these categories (Supplementary Figure S6). All together, the results suggested that, regardless of the variation in waterlogging tolerance among plant species, common responses could still be identified.

**Figure 7:**
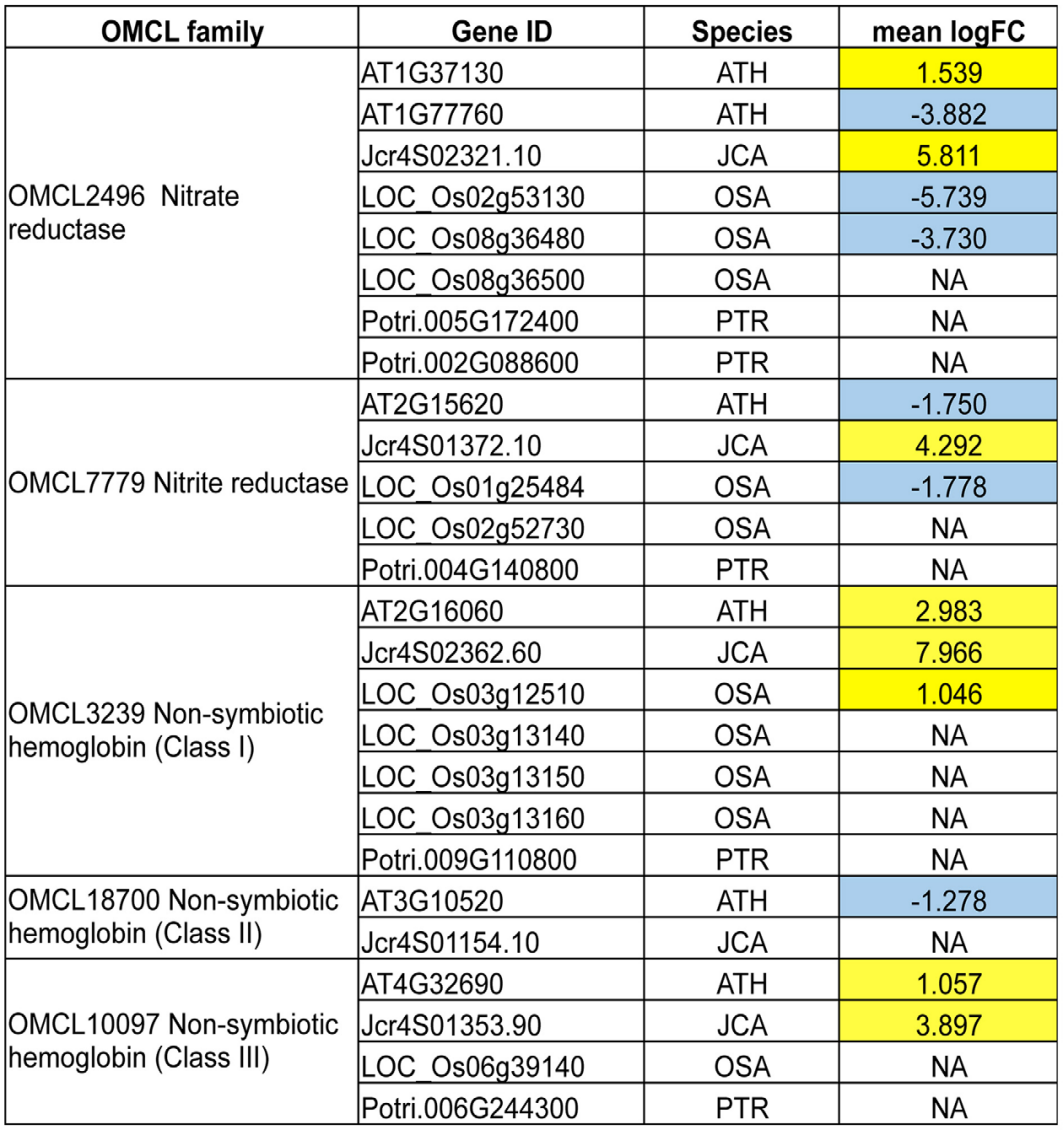
**Response of OMCL clusters with genes involved in nitrogen metabolism and NO production to waterlogging**. Yellow, Up-regulated DEGs; Blue, Down-regulated DEGs; White, Orthologs not differentially expressed. Numbers represent mean log_2_ fold change values.

To examine whether there is a link between tolerance levels and specific molecular mechanisms, we further inspected the PAGEMAN ORA results. We found that multiple processes were specifically regulated in Jatropha. For example, the genes involved in non-mevalonate pathway biosynthesis of isoprenoids, response to stress including heat and touch/wounding, and calcium signaling were up-regulated, while lipid metabolism, cold stress response, and cell organization were down-regulated (Figure 6). In rice, we found several protein kinases genes were specifically up-regulated (Figure 6). Interestingly, we also found that the down-regulation of genes controlling NO^−^_3_ metabolism, including NR, was specific to rice (Figure 6). An OMCL cluster analysis also confirmed that, while NRs were specifically down-regulated in rice, this was not the case in Arabidopsis and Jatropha (Figure 7). Additionally, the analysis also showed that up-regulation of NIR was specific to Jatropha. Evidently, the up-regulation of class I *nsHbs* (OMCL3239), putative NO scavengers, was found in Arabidopsis, Jatropha, and rice. However, the induction levels were much higher in Jatropha than in Arabidopsis and rice (Figure 7).

Comparative analysis of TFs in response to waterlogging revealed that the expression pattern of TFs was generally conserved in all plant species examined (Supplementary Table S5). OMCL clusters of ERFs, bZIPs, NACs and WRKYs were induced by waterlogging. Interestingly, OMCL clusters of trihelix TFs were found to be up-regulated in Arabidopsis, Jatropha, and gray poplar but not in rice (Supplementary Table S5). For protein kinases, OMCL clusters of leucine rich repeat kinases XI and XII and wall-associated kinases were induced in much greater quantities in rice (Supplementary Table S5). Examination of OMCL clusters of heat stress and redox regulation revealed that the induction of alternative oxidase was found only in Jatropha. Up-regulation of heat-shock proteins (HSPs), were commonly found in both intolerant and tolerant species (Supplementary Table S5). Interestingly, OMCL clusters of peroxidases were greatly induced in rice (Supplementary Table S5).

## Discussion

In this study, high-throughput RNA-sequencing technology was employed to compare differential gene expression profiles of Jatropha roots subjected to 24 h WS. Our study provides a new insight into understanding the molecular mechanisms underlying the response to waterlogging in Jatropha. WS reprogrammed expression of 1968 DEGs; seven of these were further confirmed to be differentially expressed by quantitative real-time PCR (Figure 5). By applying bioinformatic analyses for non-model species, our study demonstrated that a number of cellular processes in Jatropha roots were affected by WS. These included anaerobic fermentation, carbohydrate metabolism, glycolysis, ethylene synthesis and perception, NO^−^_3_ metabolism, NO production, regulation of transcription, protein degradation, transport, and signaling, suggesting that diverse physiological processes were affected by WS.

Plants subjected to low oxygen conditions shift their metabolism from oxidative phosphorylation to anaerobic fermentation to maintain ATP production (Bailey-Serres et al., 2012). Our results supported a longstanding notion that waterlogging promotes anaerobic respiration as observed by the up-regulation of DEGs encoding enzymes in glycolysis and fermentation (Figure 3; Supplementary Table S1). Remarkably, the expression of *Alternative oxidase* (*AOX*) was specifically enhanced in roots of waterlogged Jatropha (*Jcr4S02312.80*, Supplementary Table S1) implying that AOX may function as an alternative to cytochrome oxidase under low oxygen conditions. The increase in *AOX* gene expression could prevent reactive oxygen species (ROS) formation from the over-reduction of the ubiquinone pool. Plants can initiate additional responses to low oxygen conditions, including down-regulation of energy consuming processes such as storage metabolism (Geigenberger et al., 2000) and switching from invertase to sucrose synthase for hydrolysis of sucrose (e.g., Guglielminetti et al., 1995, 1997; Biemelt et al., 1999; Zeng et al., 1999; Albrecht and Mustroph, 2003; Bologa et al., 2003; Bieniawska et al., 2007). Here, we observed in the roots of waterlogged Jatropha, the down-regulation of genes involved in the synthesis of the cell wall, DNA, secondary metabolites, and starch (Figure 2) and the up-regulation of sucrose synthase (Figure 3A). These results, together with the finding that waterlogging resulted in the decrease in total root carbohydrate (Supplementary Figure 1B) provided a strong conclusion that Jatropha roots responded to waterlogging via the regulation of energy consumption and production.

Low oxygen conditions in plants promote the utilization of NO^−^_3_ and the production of NO to facilitate anaerobic survival (Horchani et al., 2010; Oliveira et al., 2013; Oliveira and Sodek, 2013). Alternative to anaerobic fermentation, NO^−^_3_ could be used as NADH acceptors. This allows NAD^+^ to be reused in glycolysis. It has been observed that longer periods of low oxygen survival can be achieved when NO^−^_3_ is provided (Allegre et al., 2004; Horchani et al., 2010). In waterlogged Jatropha roots, the expression of *NR*, *NiR*, and *nitrate transporter* was induced (Figure 4B; Supplementary Table S1), emphasizing the roles of NO^−^_3_ in waterlogging acclimation. Future studies should focus more on investigating the significant of NR, NiR, and NO^−^_3_ on tolerance to waterlogging in Jatropha.

Accumulation of the gas hormone ethylene is extremely important for the induction of plant responses to low oxygen. Since waterlogging inhibits gas diffusion, ethylene can be trapped in the waterlogged plant organs. Our transcriptome data revealed that root waterlogging promoted mRNA accumulation of two key enzymes in ethylene biosynthesis, *ACS* and *ACO* (Figure 3C; Supplementary Table S1). Concomitantly, the expression of *ETR*, was enhanced in waterlogged Jatropha roots. These findings suggested that waterlogging might increase ethylene synthesis and perception in Jatropha roots. Our study recognized three group-VII ERFs from the Jatropha genome. Two of these, designated JcERFVII-2 and JcERFVII-3, were noticeably induced in response to waterlogging (Figures 4, 5; Supplementary Table S1). Previous studies identified the two group-VII ERF genes, *Snorkel* and *Sub1*, as key players in the submergence response in deepwater rice and lowland rice varieties, respectively, (Xu et al., 2006; Hattori et al., 2009). These two rice varieties utilize two contrasting strategies that allow adaptive growth responses to submergence. *Snorkel* promotes the internode elongation in the deepwater rice, whereas, *Sub1* restricts the shoot elongation in the lowland rice. In Arabidopsis, studies have also shown that the group-VII ERFs function in the low-oxygen response (Hinz et al., 2010; Gibbs et al., 2011; Licausi et al., 2011). Taken together, we propose that the group-VII ERFs are promising candidates for engineering of waterlogging tolerant Jatropha. In support of this idea, overexpression of the Arabidopsis group-VII ERFs (HRE1, HRE2, RAP2.2, and RAP2.12) significantly improved low oxygen survival by promoting expression of the genes involved in low oxygen adaptation (Hinz et al., 2010; Gibbs et al., 2011; Licausi et al., 2011).

Enhanced NO production by low oxygen conditions has been documented in plants (Rockel et al., 2002; Dordas et al., 2003). NO is believed to be a signaling molecule. As previously mentioned, NO can be synthesized from NO^−^_3_ via NR and NiR (Stohr et al., 2001). In Arabidopsis, it was reported that accumulation of NO could induce *AOX* expression via inhibition of aconitase resulting in accumulation of citrate and a shift of metabolism toward nitrogen assimilation under hypoxia (Gupta et al., 2012). A recent study revealed that NO could control the stability of Arabidopsis group-VII ERFs via proteolytic modulation (Gibbs et al., 2014). We speculate that enhanced NO accumulation in Jatropha roots under low oxygen conditions could destabilize the group-VII ERF proteins and, therefore, decrease low oxygen tolerance. Modulation of NO accumulation via nsHbs in roots under low oxygen conditions has been reported (Dordas et al., 2003, 2004; Perazzolli et al., 2004; Hebelstrup et al., 2006). Our study demonstrated that waterlogging could induce the expression of *nsHbs* in Jatropha roots (Figure 7; Supplementary Table S1). The function of nsHbs possibly involves fine-tuning the accumulation of NO in waterlogged roots of Jatropha. Future research to improve waterlogging tolerance should emphasize genetic engineering to modulate NO levels.

Nearly six percent of DEGs encoding for TFs was identified in this study, implying that transcriptional regulation plays an important role in the waterlogging response. In agreement with the finding that ethylene synthesis and perception were activated, this study recognized AP2/ERF TFs as the most pronounced TFs in response to waterlogging (Figure 4A). Waterlogging induced AP2/ERFs included two of group-VII ERFs and an additional 19 ERF, whose function might be associated with waterlogging acclimation (Supplementary Table S1). The ERF IX group was the most represented waterlogging-induced ERFs in this study (Supplementary Table S1). The function of the group-IX ERFs was associated with hormonal response, such as the responses to ethylene (Wang et al., 2007) or ethylene in combination with jasmonate (Champion et al., 2009). The induction of *JcERFX-6* (*Jcr4S03895.40*; OMCL 2117), an ortholog of Arabidopsis *RAP2.6L*, was evident in this analysis (Supplementary Table S1). Overexpression of *RAP2.6L* in Arabidopsis improved the waterlogging response by delaying waterlogging-induced premature senescence (Liu et al., 2012). In this study, the expression of *trihelix TF* (*Jcr4S02762.30*: OMCL11085), an ortholog of Arabidopsis *HYPOXIA RESPONSE ATTENUATOR1* (*HRA1*), was found to be strongly up-regulated by waterlogging (Supplementary Table S1). Recently, Giuntoli et al. (2014) demonstrated that HRA1 negatively regulates RAP2.12 through protein–protein interaction and proposed that the modulation of the aerobic response by group-VII ERFs and HRA1 is important to control plant responses to low oxygen stress. In addition, our analysis demonstrated that waterlogging enhanced the expression of several members of MYBs and WRKYs. An in-depth functional analysis of waterlogging-induced TFs is required to further identify candidate genes for growth improvement of waterlogged Jatropha.

Previous studies reported the use of comparative transcriptome comparison for identification of conserve and species-specific responses to low oxygen stress (Mustroph et al., 2010; Narsai et al., 2011; Narsai and Whelan, 2013). In this study, comparative transcriptome analysis between Jatropha and other plant species identified a common response to waterlogging including the up-regulation of carbohydrate cleavage, glycolysis and fermentative genes and the down-regulation of genes involved in cell-wall and secondary metabolite biosynthesis (Figure 6; Supplementary Table S5). These findings also confirmed previous reports that the alteration of carbohydrate metabolism appeared to be a common response to low oxygen stress in plants. Additionally, Arabidopsis, gray poplar and Jatropha showed the up-regulation of genes involved in ethylene production and signaling (Figure 6; Supplementary Table S5), which were commonly found in response to low oxygen in several plants. The up-regulation of AP2/ERF genes by waterlogging was commonly identified in all species examined (Figure 6; Supplementary Table S5). These results strongly emphasized the roles of AP2/ERF genes in low oxygen adaptation. While comparative analysis exposed the common response to waterlogging in plants, the analysis showed that various processes were specifically regulated in each species. Contrary to what has been observed in waterlogged Jatropha, the expression of *NR* was down-regulated in submerged rice (Figure 7) suggesting the down-regulation of the *NR* gene may be a characteristic of waterlogging-tolerant species.

Global warming results in extreme climates, such as drought, flooding and heat stress. Improvement of combined stress tolerance could benefit crop production. It is widely known that stresses including drought, flooding, and high temperatures, could trigger accumulation of ROS, which causes oxidative damage of cellular components. On the other hand, ROS serves as a critical signaling molecule in the oxidative stress response. Pucciariello et al. (2012) demonstrated a connection in Arabidopsis between redox sensing and low oxygen response of seedlings. Evidence from the study of Fukao et al. (2011) showed that submergence-tolerant rice overexpressing *Sub1A* gene exhibited drought-tolerance phenotypes through oxidative stress regulation. Additionally, Loreti et al. (2005) found that several HSPs were induced by low oxygen stress and heat pretreatment enhanced anoxia tolerance of seedlings. Later on, Banti et al. (2008) demonstrated a correlation between the up-regulation of HSP mRNAs and enhanced low oxygen tolerance in Arabidopsis. These findings highlighted the roles of HSPs and ROSs in plant low oxygen acclimation. In this study, we found that waterlogging induced several OMCL clusters of HSPs and ROS enzymes in the roots of Jatropha (Supplementary Table S5). Taken together, it is possible that genetically engineered, waterlogging-tolerant crops might confer multiple stress tolerance phenotypes.

In summary, Jatropha orchestrates a complex transcriptional adjustment in response to WS that yields changes in its root physiological response to WS. This study highlights several possibilities for future investigation, including the roles of specific pathways and genes involved in the waterlogging response. Thus, a detailed characterization of individual genes must be carried out as the first step to understand their specific functions. Due to the high similarity among global *J. curcas* accessions, as reported by Popluechai et al. (2009), the improvement of waterlogging-tolerant Jatropha through traditional breeding, can be difficult to achieve. With biotechnology advances, genetic engineering and gene transformation into Jatropha are now feasible (Mukherjee et al., 2011). A recently developed protocol by Jaganath et al. (2014) enables Agrobacterium-mediated in planta transformation into Jatropha that does not require *in vitro*-based multiplication of transformed plants. Clearly, this method has the potential to facilitate the genetic modification of Jatropha. Successfully validated candidate genes could be targeted for the engineering of waterlogging-tolerant Jatropha.

### Conflict of interest statement

The authors declare that the research was conducted in the absence of any commercial or financial relationships that could be construed as a potential conflict of interest.

## Supplementary material

Supplementary Figure S1 **Physiological responses of Jatropha seedlings to waterlogging. (A)** Total leaf chlorophyll content. Data represent mean ± SE (*n* = 6). **(B)** Total root carbohydrate content. Data represent mean ± SE (*n* = 3). Letters represent significant differences calculated by One-Way ANOVA (*p*-value < 0.05).

Supplementary Figure S2 **Change in the expression of selected fermentative genes in response to waterlogging in leaves and roots of Jatropha**. Semi-quantitative PCR analysis of fermentative genes (*ADHs* and *PDC*) from 24-h waterlogged (WS) and non-stressed (NS) Jatropha roots and leaves.

Supplementary Figure S3 **Statistic and quality control of RNA-seq data. (A)** Mapping statistics **(B)** Pearson's correlation derived from CPM values. **(C)** A multi-dimension scaling plot of gene expression data. NSR, non-stressed root sample; WSR, waterlogged root sample. Numbers represent numbers of replicates.

Supplementary Figure S4 **Differentially regulated OMCL clusters grouped by the number of organisms per cluster**.

Supplementary Figure S5 **Waterlogging responsive OMCL clusters containing “49 core hypoxia” genes (Mustroph et al., 2009, 2010)**. Arabidopsis (ATH), Jatropha (JCA), gray poplar (PTR), and rice (OSA). Numbers indicated numbers of induced genes found in clusters. Yellow, induced orthologs; White, no induced orthologs; Gray (NA), no orthologs identified.

Supplementary Figure S6 **OMCL clusters with genes involved in glycolysis and fermentation**. OMCL clusters with change in at least one species were included. Up, Up-regulated DEGs; Down, Down-regulated DEGs. Bright yellow, Cluster has >1 induced DEG; Light yellow, Cluster has 1 induced DEG; Bright blue, Cluster includes >1 reduced DEG in; Light blue, Cluster includes 1 reduced DEG; White, No DEG ortholog identified; Gray, ortholog not identified.

Supplementary Table S1 **Analysis of differentially regulated genes**.

Supplementary Table S2 **A list of Jatropha AP2/ERF transcription factors**.

Supplementary Table S3 **Real-time PCR primer information**.

Supplementary Table S4 **Analysis of waterlogging transcriptome from Jatropha, Arabidopsis, gray poplar, and rice**.

Supplementary Table S5 **Comparative analysis of waterlogging transcriptome**.
