# Supplementary figures and images for: Elucidation of the molecular responses to waterlogging in Jatropha roots by transcriptome profiling
Source: Front Plant Sci. 2014 Dec 2;5:658. doi: 10.3389/fpls.2014.00658 (PMC4251292; doi:10.3389/fpls.2014.00658)

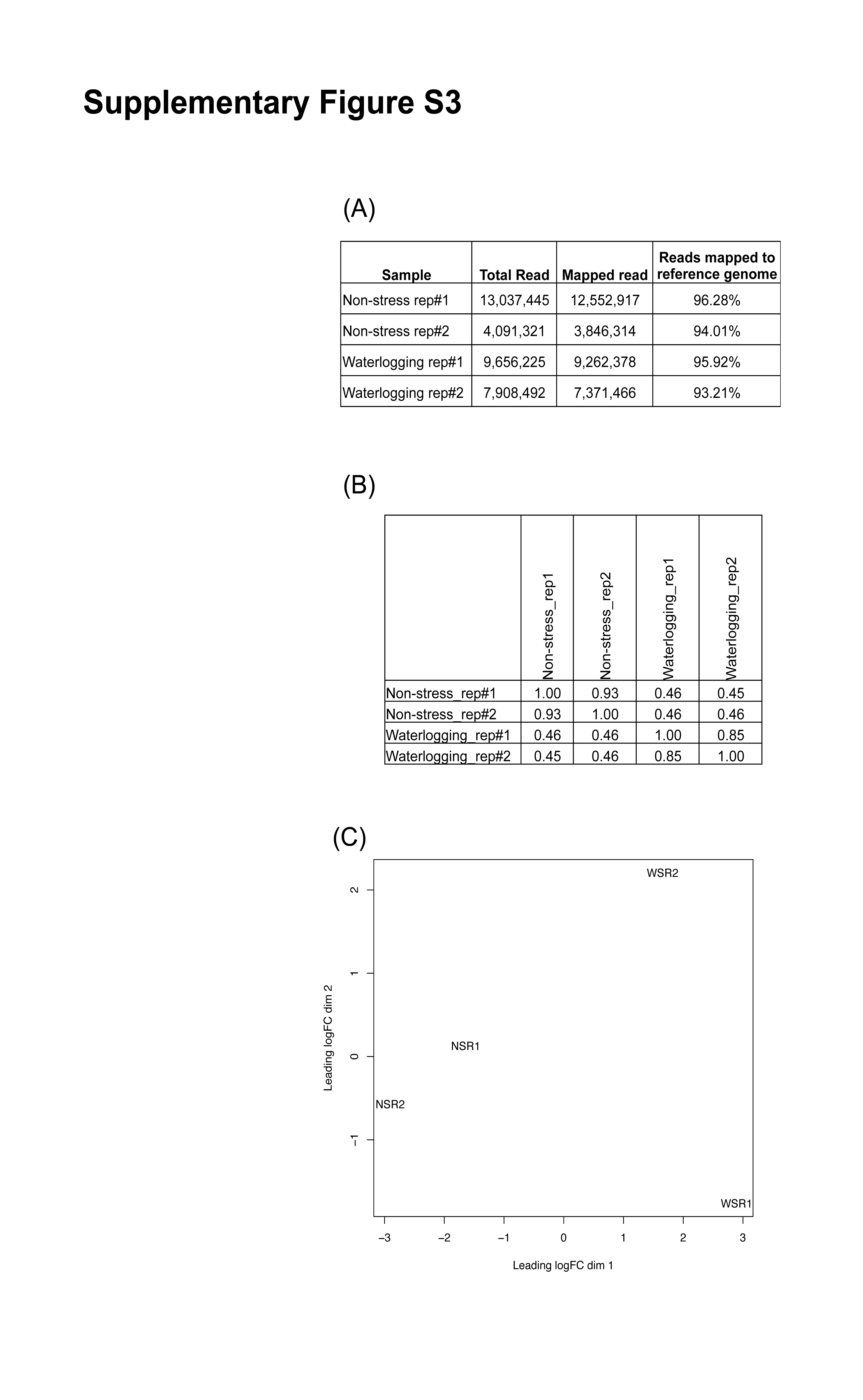

Supplement: Supplementary Figure S1 — Physiological responses of Jatropha seedlings to waterlogging. (A) Total leaf chlorophyll content. Data represent mean ± SE (n = 6). (B) Total root carbohydrate content. Data represent mean ± SE (n = 3). Letters represent significant differences calculated by One-Way ANOVA (p-value < 0.05). [file DataSheet1.ZIP › S3.TIF]

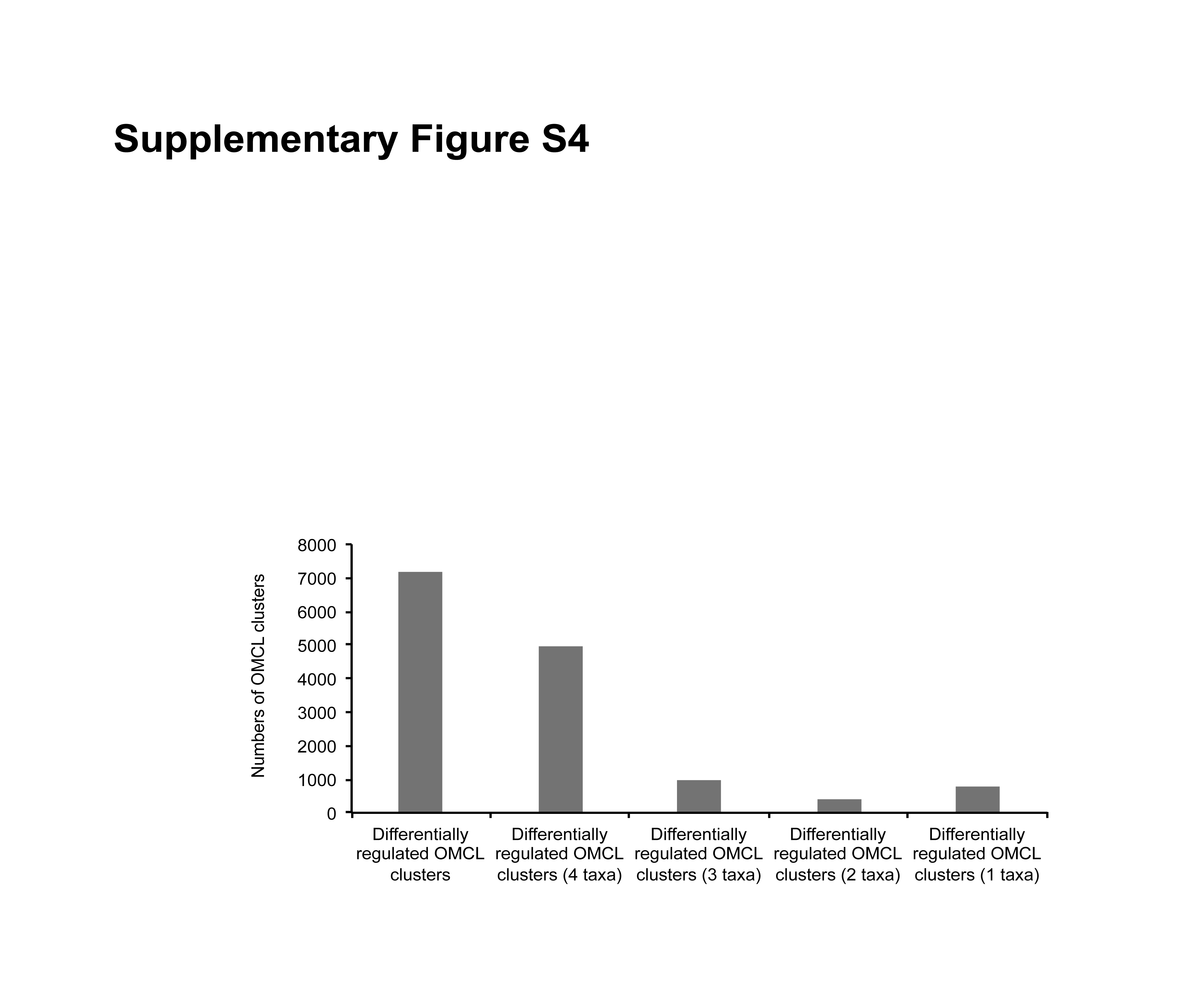

Supplement: Supplementary Figure S1 — Physiological responses of Jatropha seedlings to waterlogging. (A) Total leaf chlorophyll content. Data represent mean ± SE (n = 6). (B) Total root carbohydrate content. Data represent mean ± SE (n = 3). Letters represent significant differences calculated by One-Way ANOVA (p-value < 0.05). [file DataSheet1.ZIP › S4.TIF]

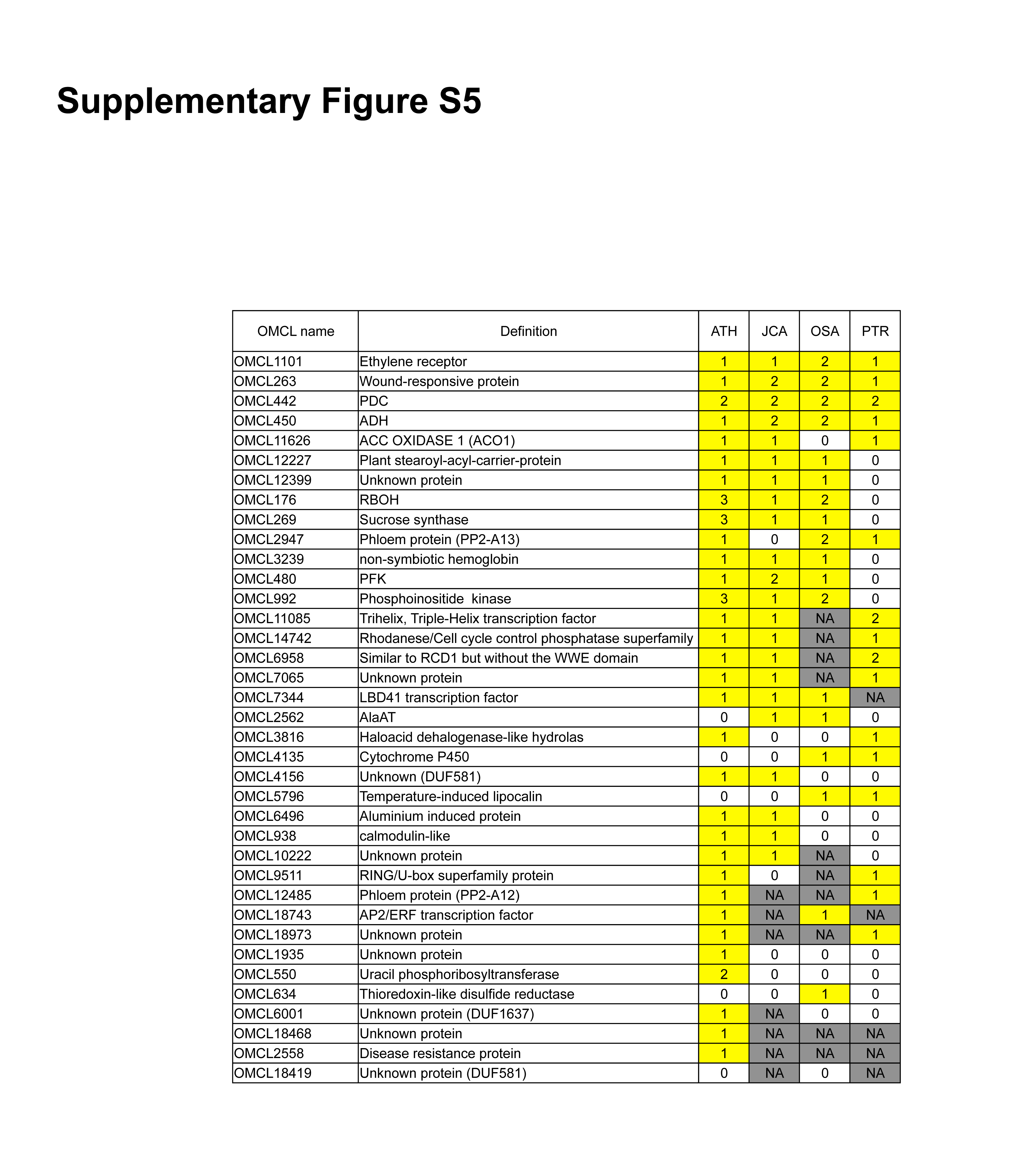

Supplement: Supplementary Figure S1 — Physiological responses of Jatropha seedlings to waterlogging. (A) Total leaf chlorophyll content. Data represent mean ± SE (n = 6). (B) Total root carbohydrate content. Data represent mean ± SE (n = 3). Letters represent significant differences calculated by One-Way ANOVA (p-value < 0.05). [file DataSheet1.ZIP › S5.TIF]

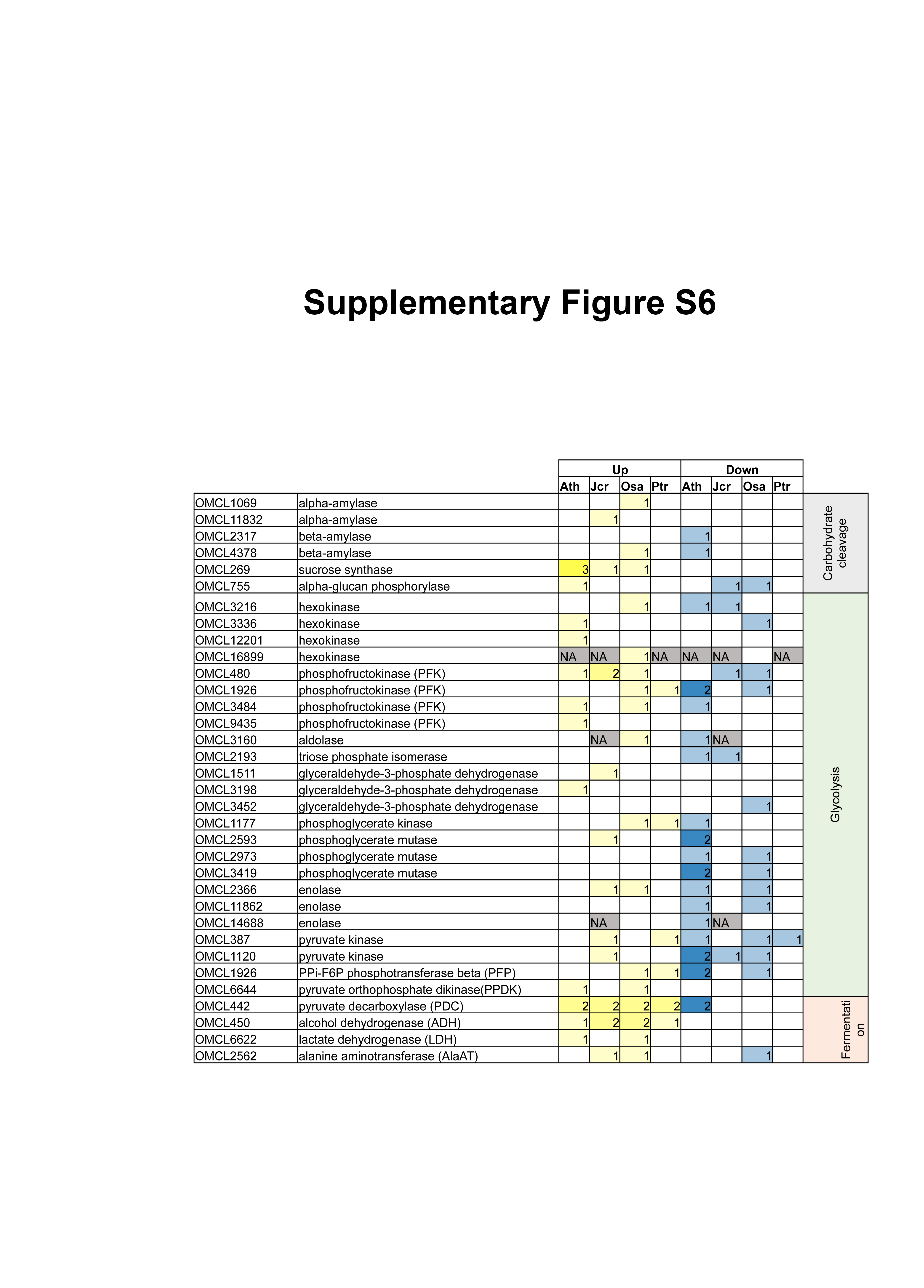

Supplement: Supplementary Figure S1 — Physiological responses of Jatropha seedlings to waterlogging. (A) Total leaf chlorophyll content. Data represent mean ± SE (n = 6). (B) Total root carbohydrate content. Data represent mean ± SE (n = 3). Letters represent significant differences calculated by One-Way ANOVA (p-value < 0.05). [file DataSheet1.ZIP › S6.TIF]

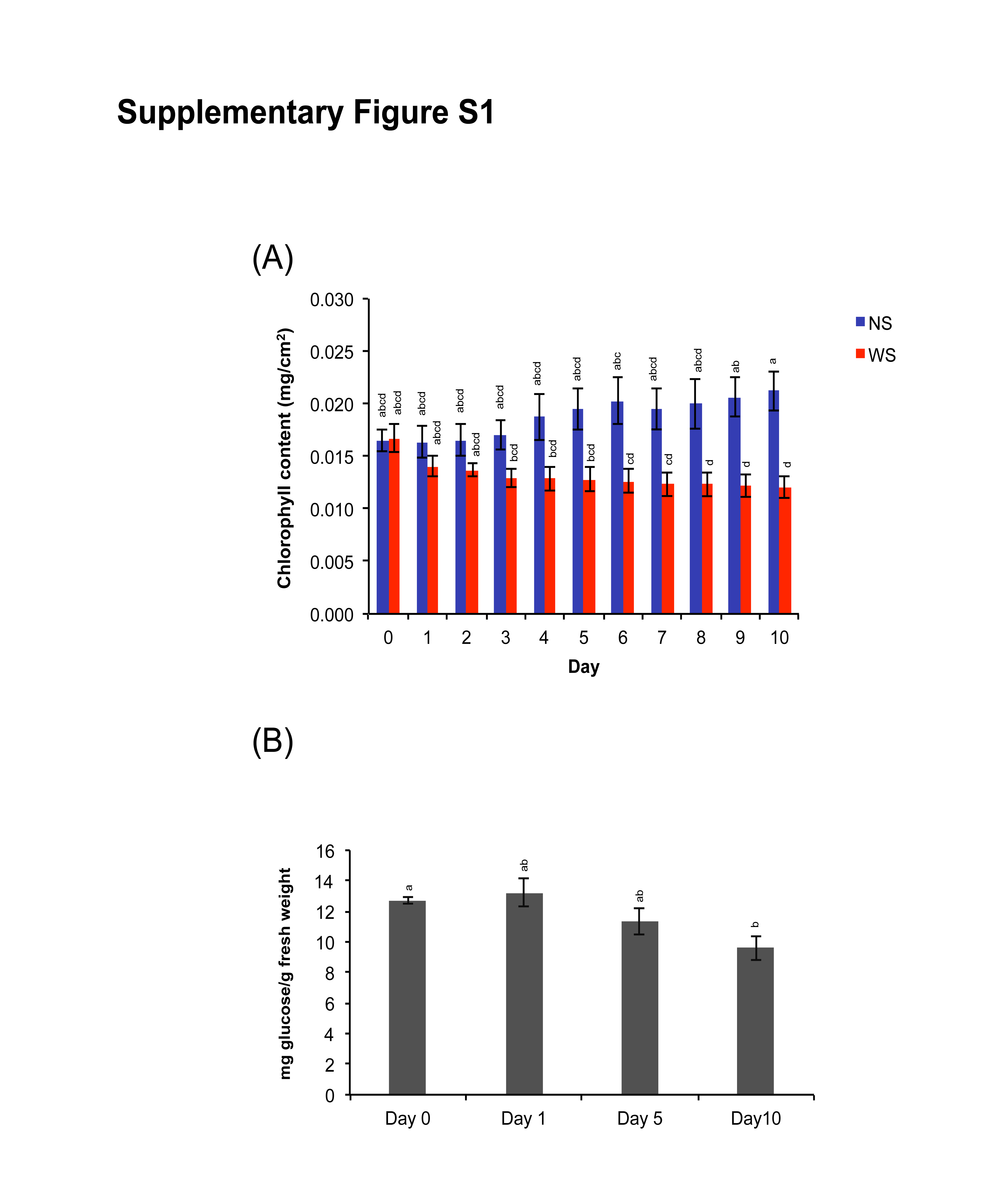

Supplement: Supplementary Figure S1 — Physiological responses of Jatropha seedlings to waterlogging. (A) Total leaf chlorophyll content. Data represent mean ± SE (n = 6). (B) Total root carbohydrate content. Data represent mean ± SE (n = 3). Letters represent significant differences calculated by One-Way ANOVA (p-value < 0.05). [file DataSheet1.ZIP › S1.TIF]

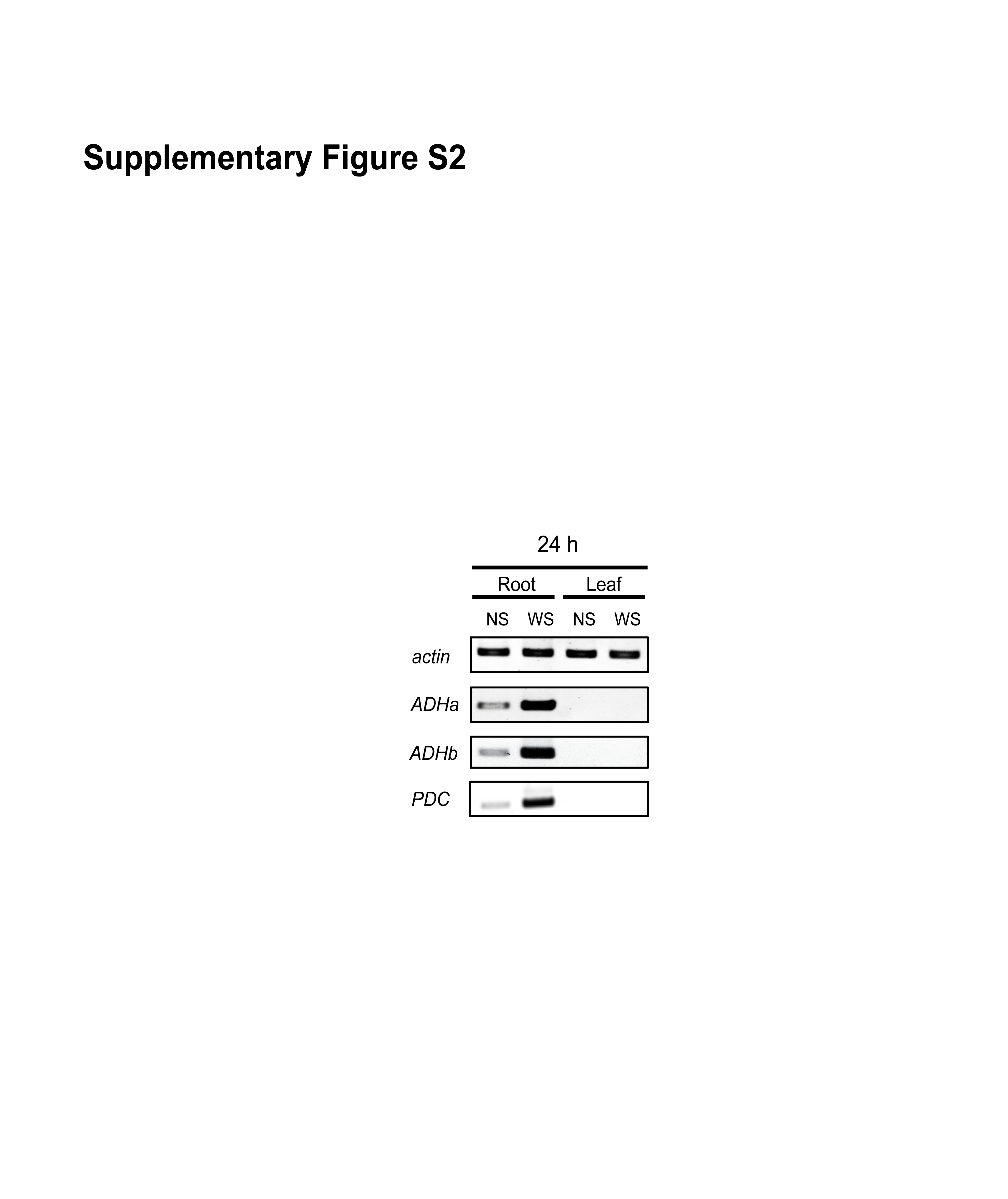

Supplement: Supplementary Figure S1 — Physiological responses of Jatropha seedlings to waterlogging. (A) Total leaf chlorophyll content. Data represent mean ± SE (n = 6). (B) Total root carbohydrate content. Data represent mean ± SE (n = 3). Letters represent significant differences calculated by One-Way ANOVA (p-value < 0.05). [file DataSheet1.ZIP › S2.TIF]
